# Algorithm for DNA sequence assembly by quantum annealing

**DOI:** 10.1186/s12859-022-04661-7

**Published:** 2022-04-07

**Authors:** Katarzyna Nałęcz-Charkiewicz, Robert M. Nowak

**Affiliations:** Institute of Computer Science, Warsaw University of Technology, Warsaw, Poland

**Keywords:** De novo assembly, Quantum annealing, Hybrid algorithm, Travelling salesman problem, TSP, Vehicle routing problem, VRP

## Abstract

**Background:**

The assembly task is an indispensable step in sequencing genomes of new organisms and studying structural genomic changes. In recent years, the dynamic development of next-generation sequencing (NGS) methods raises hopes for making whole-genome sequencing a fast and reliable tool used, for example, in medical diagnostics. However, this is hampered by the slowness and computational requirements of the current processing algorithms, which raises the need to develop more efficient algorithms. One possible approach, still little explored, is the use of quantum computing.

**Results:**

We present a proof of concept of de novo assembly algorithm, using the Genomic Signal Processing approach, detecting overlaps between DNA reads by calculating the Pearson correlation coefficient and formulating the assembly problem as an optimization task (Traveling Salesman Problem). Computations performed on a classic computer were compared with the results achieved by a hybrid method combining CPU and QPU calculations. For this purpose quantum annealer by D-Wave was used. The experiments were performed with artificially generated data and DNA reads coming from a simulator, with actual organism genomes used as input sequences. To our knowledge, this work is one of the few where actual sequences of organisms were used to study the de novo assembly task on quantum annealer.

**Conclusions:**

Proof of concept carried out by us showed that the use of quantum annealer (QA) for the de novo assembly task might be a promising alternative to the computations performed in the classical model. The current computing power of the available devices requires a hybrid approach (combining CPU and QPU computations). The next step may be developing a hybrid algorithm strictly dedicated to the de novo assembly task, using its specificity (e.g. the sparsity and bounded degree of the overlap-layout-consensus graph).

## Background

Assembling fragments of DNA sequences is an important phase in reproducing the complete genome sequences of the studied organisms. Particularly noteworthy is the de novo assembly task, where there is no reference genome. This task is further complicated by the presence of reading errors and the presence of repetitive regions. With the dynamic development of third-generation sequencing (NGS) methods and the associated significant decrease in sequencing costs, the burden of the complexity of the entire process of whole-genome sequencing has shifted from biotechnology to the computational phase. Currently, the bottleneck is the acquisition of DNA readings in the sequencing process and their computer analysis. Hence the need to develop new processing algorithms, with particular emphasis on their efficiency. In addition to the well-known and used methods of parallelizing computations through GPUs, dedicated FPGAs, or large computing clusters, one of the possible paths is to use the potential of quantum computers.

Existing de novo assembly tools are based on two algorithmic solutions: de Bruijn and overlap-layout-consensus graphs. The techniques used vary with the length of the reads. Hybrid approaches are also being developed, using both short and long reads [1]. One possible approach to assembling sequences without a reference genome is to formulate the problem as an optimization task, namely the travelling salesman problem (TSP). This issue, formulated in its general form in the 1930s by Karl Menger, is one of the best known combinatorial problems. Its main difficulty is that the increase in the number of possibilities to be considered with the increasing size of the input data is factorial. It has been proven that TSP belongs to the class of NP-hard problems [2], which makes it an interesting problem from an algorithmic point of view and causes that, although it can be considered one of the best-studied combinatorial problems in computer science, there are still attempts to develop more efficient algorithms to solve it. In its classic form, the task is to find the optimal (shortest) path between cities from a given set, such that each city is visited exactly once. The path has to start and end in the same city. The travelling salesman problem is used in several areas, from the most obvious, such as mapping the optimal routes for vehicles, to drilling printed circuit boards or picking orders in [3] warehouses. In bioinformatics itself, many TSP applications can be mentioned, such as multiple sequence alignment, construction of phylogenetic trees or protein structure prediction [4].

The use of quantum annealer, a device dedicated to solving optimization problems, seems to be one of the obvious directions to be checked. The principle of its operation has been described i.a. in [5]. The annealer itself works by generating independent samples by slowly transforming the initial state into a random end state taking into account a given objective function. In the conventional approach, we use thermal fluctuations, while the quantum annealer uses quantum fluctuations to transcend individual states.

The usefulness of QA has been tested, i.a. in [6]. The authors analyze the capabilities and limitations of QA, such as the relatively small number of qubits and their poor conectivity. Introducing a new synthetic class of problems to take advantage of quantum tunneling effect and comparing the time of their solution achieved by the classical and D-Wave algorithms, they prove the superiority of the latter. Another example is the work [7], where the authors compare the operation of quantum annealer with the simulated annealing algorithm, as well as with the quantum Monte Carlo algorithm (where the quantum tunneling phenomenon is emulated on the CPU), demonstrating experimentally the superiority of QA for selected classes of problems. Whether QA is able to lower the asymptotic complexity of NP-hard problems in general remains an open question.

Attempts to solve TSP, or its generalized form—VRP—by means of quantum annealer have already been made, among others in the works of [8, 9]. This work is not the first attempt to use the QA in the de novo assembly problem—the topic was taken up, among others, by Boev et al. [10] and Sarkar et al. [11].

QA are a class of quantum computers based on the heuristic optimization method (the phenomenon of quantum annealing) to solve optimization and sampling problems using quantum physical systems. The most famous devices of this class are QA from D-Wave. The largest quantum computer it offers now is Advantage, with a QPU consisting of over 5000 quantum bits; however, in the experiments carried out as part of the described research, the quantum annealer in older architecture, namely D-Wave 2000Q, was used.

This study aimed to investigate the possibility of using a QA in a de novo assembly task, formulated as an optimization problem (travelling salesman problem). For this purpose, the algorithm proposed by Jugas et al. [12] for the detection of overlaps in readings of DNA sequences and their ordering was first verified by performing calculations on a classical computer. Then, the results obtained by the classical algorithm were compared with the results obtained by using the hybrid algorithm to determine the travelling salesman path (combining calculations performed on the CPU and the QPU).

## Methods

### Preliminary assumptions

**Fig. 1:**
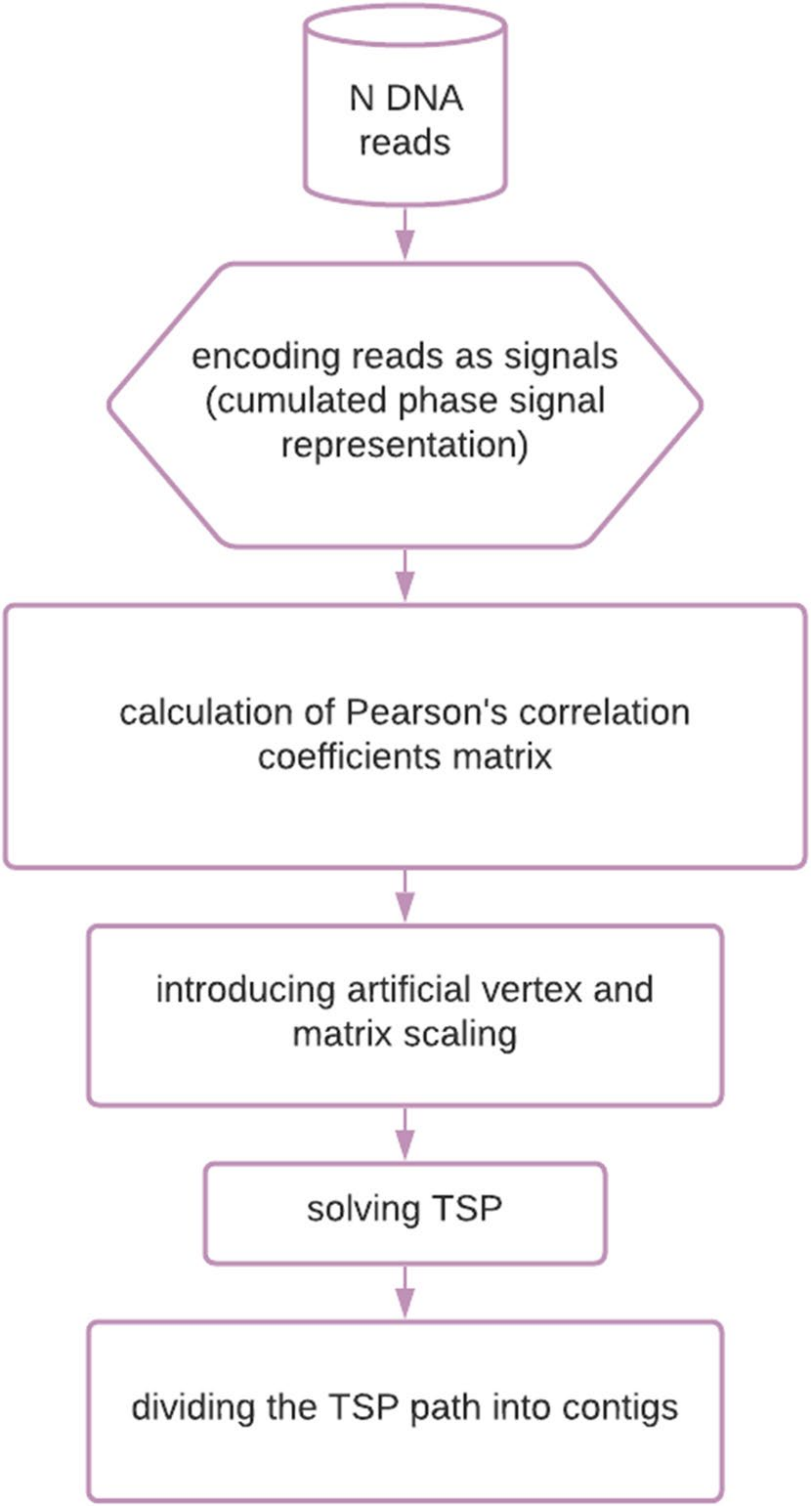
Diagram showing the steps of the algorithm

We made the following initial assumptions:

- only long reads are considered (single-end, not reversed - every read is forward strand);
- the algorithm does not take into account dealing with repetitive DNA regions;
- the tested DNA sequence fragments contain a small percentage of errors (maximum 1.5%);

The individual steps of the algorithm are presented in Fig. 1.

### Encoding DNA reads as a signal

The input of the algorithm is the set of *N* reads, a read is string over { A, C, G, T }. The first step of the algorithm is to encode the read into a signal i.e. sequence of numbers. Following the approach described by [12], cumulated phase signal representation [13] was used. According to the mapping, each letter was assigned one of the complex numbers: A: $1 + j$, C: $-1-j$, G: $-1 + j$, T: $1-j$. Then a complex number argument is calculated and values obtained in this way are accumulated in the signal. The advantage of such a representation is the preservation of information about the chemical and structural properties of the sequences, as well as the positional information, which makes it possible to compare them with each other [14].

### Pearson’s correlation coefficient matrix

The next step of the algorithm is to compare each pair of reads to detect possible overlap. The method of computing the similarity of a pair of reads, as summarized below, is taken from [12]. The Pearson correlation coefficient [15] was adopted as the measure of similarity:

1 $$\begin{aligned} \rho _{X, Y}=\frac{cov(X, Y)}{\sigma _{X}\sigma _{Y}} \end{aligned}$$

where *X*, *Y* are compared signals, *cov* is covariance and $\sigma$ stand for standard deviation. The values of the coefficient fall within the range $\left\langle -1, 1 \right\rangle$, with values close to 1 and $-1$ representing a linear relationship (positive or negative, respectively), while values close to 0 indicate a low relationship between the studied variables.

**Fig. 2:**
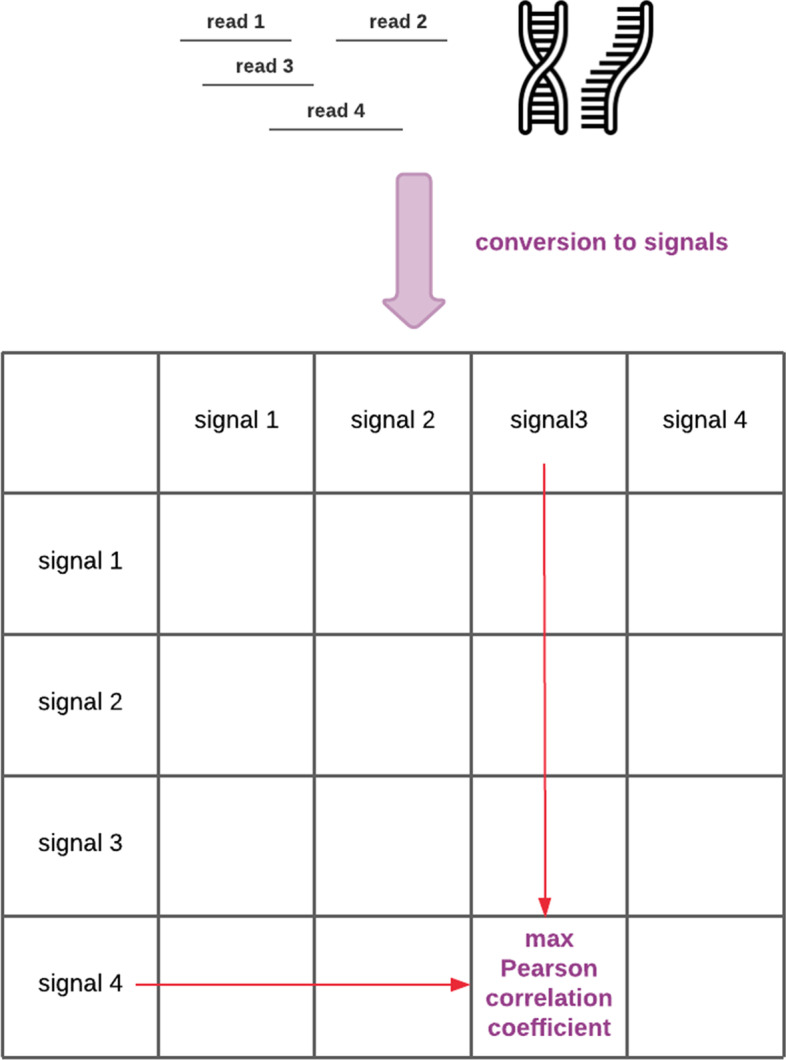
The method of calculating the Pearson correlation coefficients matrix

Given a pair of reads to compare, we shift them successively against each other, computing the Pearson correlation coefficient for overlapping sequence fragments for each shift. Then we place the maximum value obtained in this way in the resulting matrix (see Fig. 2).

### Solving travelling salesman problem

The symmetric matrix of correlation coefficients of size $N \times N$ created by this method, where *N* is the power of the input data set, is the input for the next processing step: ordering overlapping reads by solving the travelling salesman problem. The “cities” visited by the travelling salesman (the vertices of the complete undirected graph) are, in this case, the individual reads. Finding the optimal path in the graph—with the above-mentioned assumptions—allows ordering overlapping fragments of the DNA sequence and is equivalent to solving the assembly task.

### Introducing artificial vertex and scaling

In the classic formulation of the TSP, the travelling salesman starts from a specific starting point—we do not have one here. Potentially each of the reads may be the beginning or the end of the genome sequence. This difference is pointed out by Parsons et al. [16], who are therefore critical of the idea of using TSP as a solution to the de novo assembly problem. However, as Mallen-Fullerton and Anaya [17] rightly point out, this limitation is easy to circumvent by introducing an artificial vertex into the graph with zero distance from other vertices. In the algorithm that is the subject of this article, it will always be vertex marked with the number zero. Then the artificial vertex should be specified as the starting point. Thus, the Hamilton cycle obtained at the algorithm’s output, after removing the artificial vertex, will allow obtaining the searched path. Moreover, since the classical TSP minimizes the cost function, and due to the requirement to operate on integers, imposed by the Google OR Tools tool (see next paragraph), it was necessary to rescale the correlation coefficient matrix by subtracting all values from 1, multiplying by 1000 and applying the min-max scaling.

### Google OR Tools

In the work [12], which is the starting point for the described research, due to the small size of the input data, the nearest neighbour algorithm was used to solve the TSP problem. In this paper, however, to make the solution more universal, in the case of the base solution (on a classic computer), we decided to use the Google OR Tools (GOT) [18] tool. It is open-source software designed to solve combinatorial optimization problems, such as vehicle routing, scheduling or bin packing. A particular emphasis was placed on its performance—for example, on a typical computer, solving the TSP problem for 280 cities takes about one second [19]. In general, the solution returned by GOT is not always optimal due to the computational complexity of the problem belonging to the NP-hard problems class. However, since in the quantum computational model described below, one should not expect precise solutions—which results from the very nature of quantum computing—this is by no means an obstacle. In the context of de-novo assembly, obtaining a sub-optimal solution to the TSP is acceptable as long as it does not result in the detection of a false overlap. The traveling salesman path provides information which reads do actually overlap, while their final ordering to obtain the result sequence requires further processing (including calculating the exact offset between reads). This goes beyond the scope of this solution, as in the Overlap Layout Consensus approach the “Consensus” step is not the goal of our research.

The presented algorithm considers the approximate nature of the solution to the travelling salesman problem, which is reflected in the next processing step—division into contigs. Before we move on to its description, let us look at the second method of solving the TSP problem on QA.

### D-Wave-VRP

The D-Wave-VRP [20] tool^1^ was used to perform the calculations based on the quantum paradigm. This part of the experiments, intended to be calculated on the QPU, was carried out on a real quantum annealer (D-Wave 2000Q). The TSP problem is a particular case of the Vehicle Routing Problem (VRP), formulated in 1959 by Dantzig and Ramser [21], where the goal is to find optimal routes for several vehicles visiting a specific set of locations. The D-Wave-VRP tool allows solving a number of general VRP variants tasks, such as Capacitated Multi-Depot Vehicle Routing Problem (CMDVRP) or without specifying vehicle capacity (). From the point of view of this paper, the problem comes down to finding the optimal route for one vehicle with no restrictions on its capacity, with one depot (which is an artificial vertex) and *N* destinations.

In order to solve the TSP problem by means of a quantum annealer, it is necessary to formulate it as an optimization problem. In D-Wave-VRP, the TSP problem formulation as QUBO (Quadratic Unconstrained Binary Optimization) is based on [22]. A detailed description of the notation, assumptions and corresponding equations can be found in sections 2.1 and 2.2 of [20]. Paraphrasing the aforementioned paragraphs, the QUBO formulation has the following form:

2 $$\begin{aligned} QUBO_{VRP}=A_{1}C+A_{2}Q \end{aligned}$$

where $A_{1}$, $A_{2}$ are constants (their values in the D-Wave-VRP tool were set as $A_{1}=1$, $A_{2}=10^{7}$). Component C of the above equation, which is the essence of VRP, has the form:

3 $$\begin{aligned} \begin{aligned} C&=\sum _{m=1}^{M}\sum _{n=1}^{N}x_{m,n,1}C_{N+1,n}+\sum _{m=1}^{M}\sum _{n=1}^{N}x_{m,n,N}C_{n,N+1}\\&\quad +\sum _{m=1}^{M}\sum _{n=1}^{N-1}\sum _{i=1}^{N+1}\sum _{j=1}^{N+1}x_{m,i,n}x_{m,j,n+1}C_{i,j} \end{aligned} \end{aligned}$$

where *M* is the number of warehouses, *N* is the number of points visited, $C_{i, j}$ is the distance from *i* to *j*, $x_{i, j, k}$ is a binary variable accepting value 1 then and only then vehicle number *i* visits vertex *j* as *k*-th on its route. The following term ensures that each point is visited by exactly one vehicle exactly once:

4 $$\begin{aligned} \begin{aligned} Q&=\sum _{k=1}^{N}A(x_{1,k,1},x_{2,k,1},\dots ,x_{1,k,2},\dots ,x_{M,k,N})\\&\quad +\sum _{m=1}^{M}\sum _{n=1}^{N}A(x_{m,1,n},x_{m,2,n},\dots ,x_{m,N+1,n}) \end{aligned} \end{aligned}$$

In this paper, we treat TSP as a special case of VRP, where the number of both vehicles and warehouses is 1. Instead of the binary variables $x_{i, j, k}$ defined above, we will continue to use the notation $x_{i, j}$, where $x_{i, j}$ is 1 then and only then the salesman visits vertex *i* as *j*-th in sequence. Since $M = 1$, we can simplify *C* to the following form:

5 $$\begin{aligned} C=\sum _{n=1}^{N}x_{n,1}C_{N+1,n}+\sum _{n=1}^{N}x_{n,N}C_{n,N+1}+\sum _{n=1}^{N-1}\sum _{i=1}^{N+1}\sum _{j=1}^{N+1}x_{i,n}x_{j,n+1}C_{i,j} \end{aligned}$$

On the other hand, term *Q*, which ensures that every point on the traveling salesman’s route is visited and that it is visited exactly once, has the form:

6 $$\begin{aligned} Q=\sum _{k=1}^{N}A(x_{k,1},x_{k,1},\dots ,x_{k,2},\dots ,x_{k,N})+\sum _{n=1}^{N}A(x_{1,n},x_{2,n},\dots ,x_{N+1,n}) \end{aligned}$$

Due to the size of the considered graphs (the described tool should enable solving TSP for $N > 50$), it was necessary to divide it into sub-problems. A problem of size *N* translates to a QUBO model of size $N^{2}$, which means that for problem $N=50$ it cannot be embedded directly into the QPU. According to [23], the largest fully connected problem that *can be embedded onto the 2000Q has approximately 60 spins, while the limit in practice depends on the number of faulty physical qubits in the device*. This means that the largest possible TSP problem to be solved directly in the 2000Q architecture is $N = 7$ (because already for $N = 8$ we have $N^{2} = 64 > 60$). It raises a necessity to decompose and use a hybrid approach.

DBScanSolver, based on the recursive DBSCAN algorithm [24], was selected from among the four possible solvers. The algorithm uses DBSCAN as clustering algorithm, where the number of clusters is limited. For each cluster TSP problem is solved separately and then results are merged. The division into sub-problems is done on the CPU, while the search for traveling salesman path for a given sub-problem is performed on QPU.

The default configuration of D-Wave-VRP tool has not been changed. The quantum annealing algorithm uses two branches—tabu search and a sample of subproblems returned from the D-Wave system that “compete” with each other; a state with minimum energy is selected. There is no maximum computation time per QPU or maximum number of iterations. The convergence parameter takes the value 1, which means that the process ends after one iteration of the calculations, during which the values obtained on the output have not changed. for every algorithm and on every test case we ran 5 experiments.

### Dividing the TSP path into contigs

Having the solution to the TSP problem—the optimal or near-optimal path passing through all the vertices in the graph—it is necessary to proceed to the last step of the algorithm, which is the contigs detection. This step is necessary since the algorithm could make a choice that was not a de facto overlap while looking for successive passes for a travelling salesman. In other words, the resulting path may combine reads that do not overlap each other. Therefore, it is necessary to detect such places and divide the path into smaller fragments, constituting a series of overlapping reads of the DNA sequence. For this purpose, calculations based on the knowledge of the degree of sequence coverage were used. For each link in the path, it is checked whether the value of the estimate function in the distance matrix exceeds a certain fixed threshold level. Based on the knowledge of coverage value *COV*, we know that a given read should coincide on average with $COV - 1$ other reads. Exceeding the threshold determined in this way is considered a break. By reducing the value of the threshold parameter, we can increase the number of contigs while reducing the probability of a false positive rate. The result of the described method is a set of contigs understood as a list of reads, each of which overlaps.

### Data preparation

The experiments were carried out for both artificially generated data (dataset A) and actual sequences of organisms with small DNA sizes (dataset B)—lambda phage (48k bp) and *E. coli* bacteria (for the first 50k bp), being widely used model organisms, as well as additionally on the SARS COVID-19 (29k bp) sequence. A summary of information about the datasets used is provided in the Table 1.

**Fig. 3:**
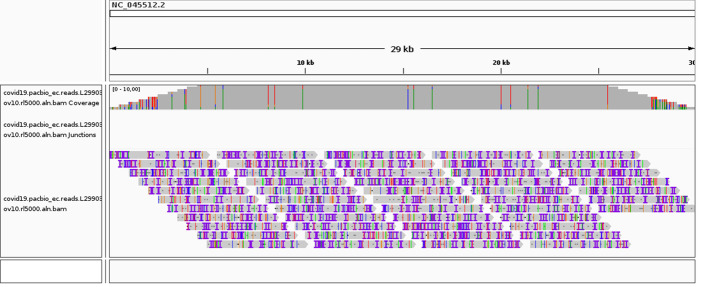
Arrangement of reads in relation to the input sequence for exemplary sequence (COVID-19) from dataset B

Random sequences were generated as follows: for different overlap values $\left\{ 2700, 2500, 2300 \right\}$, coverages$\left\{ 5, 10, 15 \right\}$ and percentage of errors $\left\{ 0, 0.5, 1, 1.5 \right\}$ a sequence was created DNA as a random string of characters from the *A*, *G*, *C*, *T* alphabet, selected with equal probability. This method of generating sequences eliminated the risk of repeated regions, to which—as indicated in section Preliminary assumptions—the discussed algorithm is not resistant. The reads length was 3000 bp (before introducing point mutations simulating errors). A total of 50 reads were generated for each sequence. A given coverage was obtained by a different length of the input sequence. It was decided to treat the starting sequence as a cyclic buffer so that the beginning and the end of the sequence would not be distinguished in any way. An exemplary set of reads from dataset A and their arrangement on the output sequence is shown in the Fig. 3.

The msbar tool from the EMBOSS [25] package was used to simulate sequencing errors, introducing a specific percentage of point mutations (insertions, deletions, substitutions, duplications) into each simulated read of a DNA sequence.

In the case of dataset B, all selected genome sequences were input for ReadSim [26]. Although this long-reads simulator, easy to install and use, is not the latest (the current version is from November 2014), it fits this paper’s needs well. In line with the assumptions mentioned in Preliminary assumptions, it was limited to single-end reads, setting a predetermined coverage value and error percentage. The lengths of the reads were generated with a uniform distribution.

**Table 1:** Parameters of sequences used for experiments

datasetA				
Overlap length	Length	Coverage	% of errors	Average read length
2300	30,000	5	0	3000
2500	30,000	5	0	3000
2700	30,000	5	0	3000
2300	30,000	5	0.5	3000
2500	30,000	5	0.5	3000
2700	30,000	5	0.5	3000
2300	30,000	5	1.0	3000
2500	30,000	5	1.0	3000
2700	30,000	5	1.0	3000
2300	30,000	5	1.5	3000
2500	30,000	5	1.5	3000
2700	30,000	5	1.5	3000
2300	15,000	10	0	3000
2500	15,000	10	0	3000
2700	15,000	10	0	3000
2300	15,000	10	0.5	3000
2500	15,000	10	0.5	3000
2700	15,000	10	0.5	3000
2300	15,000	10	1.0	3000
2500	15,000	10	1.0	3000
2700	15,000	10	1.0	3000
2300	15,000	10	1.5	3000
2500	15,000	10	1.5	3000
2700	15,000	10	1.5	3000
2300	10,000	15	0	3000
2500	10,000	15	0	3000
2700	10,000	15	0	3000
2300	10,000	15	0.5	3000
2500	10,000	15	0.5	3000
2700	10,000	15	0.5	3000
2300	10,000	15	1.0	3000
2500	10,000	15	1.0	3000
2700	10,000	15	1.0	3000
2300	10,000	15	1.5	3000
2500	10,000	15	1.5	3000
2700	10,000	15	1.5	3000

datasetB				
Sequence name	Length	Coverage	% of errors	Average read length
Covid19	29,903	10	0.33	5000
lambda_phage	48,502	10	0.33	5000
NC_000913_cov5	50,000	5	0.33	5000
NC_000913_cov10	50,000	10	0.33	5000
NC_000913_cov15	50,000	15	0.33	5000

## Results

This section presents the results of research aimed at comparing the classical algorithm implemented with the use of GOT and the algorithm implemented in a hybrid (classical and quantum) paradigm, where the quantum part was realized on the QA by D-Wave (designated as DWVRP).

### Algorithms evaluation

The developed pipeline is by no means a complete, ready-made assembler, which makes it impossible to use standard methods of comparing it with existing tools. Following [12], to evaluate the operation of the tested algorithms (classical-GOT and hybrid-DWVRP), the measure of checking the number of correctly and incorrectly detected overlaps was used. Accuracy and travelling salesman path cost were also calculated (it should be remembered that the lower the cost, the better the result). In particular, it was possible to find the optimal path with a cost of zero for error-free reads. In Tables 2, 3, 4, the *real overlaps* column shows the actual number of contigs for a given path (the independent coherent fragments into which it should be divided). This number was obtained based on the knowledge of the location of each read in the target sequence. On the other hand, the *calculated contigs* column contains the number of contigs calculated using the algorithm described in section Dividing the TSP path into contigs. It is more desirable to have a situation in which, despite the more significant number of contigs, no false overlaps are detected (false positive rate equal to zero) than the case where the number of contigs will be lower, but the result will contain a false overlap (resulting in lower reliability of individual contigs). Then, the next processing step (going beyond the scope of the described algorithm) may be combining the contigs calculated in this way, using more precise matching algorithms (e.g. Smith-Waterman algorithm). In turn, the presence of false overlaps in the results may result in errors in the final DNA sequence.

**Table 2:** Results of experiments for circular random sequences (datasetA), coverage = 5

Overlap length	Method	Path cost	Real contigs	Calculated contigs	Correct overlaps	Incorrect overlaps	Accuracy
0% errors							
2300	GOT	0	0	0	51	0	1.00
	DWVRP	268	2	2	49	0	1.00
2500	GOT	0	0	0	51	0	1.00
	DWVRP	309	2	2	49	0	1.00
2700	GOT	0	0	0	51	0	1.00
	DWVRP	113	1	1	50	0	1.00
0.5% errors							
2300	GOT	449	2	2	49	0	1.00
	DWVRP	648	1	6	45	0	0.90
2500	GOT	350	2	3	48	0	0.98
	DWVRP	390	1	7	44	0	0.88
2700	GOT	349	0	3	48	0	0.94
	DWVRP	484	0	10	41	0	0.80
1.0% errors							
2300	GOT	507	0	2	49	0	0.96
	DWVRP	965	3	6	45	0	0.94
2500	GOT	469	1	4	47	0	0.94
	DWVRP	892	3	7	44	0	0.92
2700	GOT	648	1	5	46	0	0.92
	DWVRP	881	2	8	43	0	0.88
1.5% errors							
2300	GOT	912	0	3	48	0	0.94
	DWVRP	1331	2	5	46	0	0.94
2500	GOT	1054	3	5	45	1	0.92
	DWVRP	2233	10	12	39	0	0.96
2700	GOT	724	0	8	43	0	0.84
	DWVRP	1124	2	10	41	0	0.84

**Table 3:** Results of experiments for circular random sequences (datasetA), coverage=10

Overlap length	Method	Path cost	Real contigs	Calculated contigs	Correct overlaps	Incorrect overlaps	Accuracy
0% errors							
2300	GOT	0	0	0	51	0	1.00
	DWVRP	0	0	0	51	0	1.00
2500	GOT	0	0	0	51	0	1.00
	DWVRP	65	1	1	50	0	1.00
2700	GOT	0	0	0	51	0	1.00
	DWVRP	0	0	0	51	0	1.00
0.5% errors							
2300	GOT	127	0	0	51	0	1.00
	DWVRP	176	0	1	50	0	0.98
2500	GOT	138	0	1	50	0	0.98
	DWVRP	300	1	3	48	0	0.96
2700	GOT	150	0	0	51	0	1.00
	DWVRP	196	0	1	50	0	0.98
1.0% errors							
2300	GOT	325	0	0	51	0	1.00
	DWVRP	482	0	3	48	0	0.94
2500	GOT	380	0	1	50	0	0.98
	DWVRP	719	2	6	45	0	0.92
2700	GOT	288	0	1	50	0	0.98
	DWVRP	509	1	4	47	0	0.94
1.5% errors							
2300	GOT	422	1	0	50	1	0.98
	DWVRP	578	0	4	47	0	0.92
2500	GOT	483	0	0	51	0	1.00
	DWVRP	581	0	2	49	0	0.96
2700	GOT	363	0	1	50	0	0.98
	DWVRP	636	1	3	48	0	0.96

**Table 4:** Results of experiments for circular random sequences (datasetA), coverage=15

Overlap length	Method	Path cost	Real contigs	Calculated contigs	Correct overlaps	Incorrect overlaps	Accuracy
0% errors							
2300	GOT	0	0	0	51	0	1.00
	DWVRP	0	0	0	51	0	1.00
2500	GOT	0	0	0	51	0	1.00
	DWVRP	0	0	0	51	0	1.00
2700	GOT	0	0	0	51	0	1.00
	DWVRP	0	0	0	51	0	1.00
0.5% errors							
2300	GOT	285	0	0	51	0	1.00
	DWVRP	461	0	3	48	0	0.94
2500	GOT	450	0	3	48	0	0.94
	DWVRP	574	0	3	48	0	0.94
2700	GOT	272	0	4	47	0	0.92
	DWVRP	403	0	7	44	0	0.86
1.0% errors							
2300	GOT	640	0	0	51	0	1.00
	DWVRP	779	0	3	48	0	0.94
2500	GOT	716	0	2	49	0	0.96
	DWVRP	903	0	3	48	0	0.94
2700	GOT	509	0	2	49	0	0.96
	DWVRP	816	1	10	41	0	0.82
1.5% errors							
2300	GOT	917	0	1	50	0	0.98
	DWVRP	1994	1	8	43	0	0.86
2500	GOT	789	1	0	50	1	0.98
	DWVRP	1048	0	2	49	0	0.96
2700	GOT	837	0	3	48	0	0.94
	DWVRP	1353	2	6	45	0	0.92

### Experiments for dataset A

The first part of the research was to check the algorithm’s operation for artificially generated data (random circular sequences from dataset A). The aim was to compare the results obtained by these algorithms for different overlap values, different error percentages and three different coverage values. Better results (lower path cost, fewer contigs and higher accuracy) were expected for longer overlap lengths, but no such relationship was observed. However, as expected, with the increase in errors in DNA reads, a decrease in accuracy and an increase in the number of contigs were observed. For error-free sequences, accuracy = 1 was achieved in all three cases (Tables 2, 3 and 4), with both GOT and DWVRP achieving the optimal path for the two highest coverage values (with one exception). Moreover, the dependence of the quality of the obtained results on the value of the coverage parameter is clearly visible. Also, as expected, in all cases, better results were achieved by the algorithms performed on the classic computer - only once the accuracy value dropped below 0.85. However, what is essential, the results achieved by DWVRP were not much worse.

### Experiments for dataset B

The second stage of the research was to check the operation of the GOT and DWVRP algorithms for five actual genomic sequences (covid, lambda phage and three sequences obtained in the first 50k nucleotides of the *E. coli* genome, for the same three different coverage values as in the previous study). The obtained results, presented in table 5, do not differ significantly in quality from those obtained in previous experiments—the lowest calculated accuracy value is 0.92, and only in two cases one overlap was incorrectly detected. Also, the number of fragments (contigs) into which the travelling salesman path was divided, ranging from 0 to 10 (for the case with the highest number of readings-vertices), does not differ significantly from that observed for artificial sequences, increasing—obviously—with the complexity of the graph. Similar to results obtained for dataset A, the GOT achieved better results, but here, too, the DWVRP was only slightly worse. For the last three sequences in table 5 for the GOT, a slight increase in quality can be seen with increasing coverage; for DWVRP, there is no such dependency.

**Table 5:** Results of experiments for datasetB

Sequence	Method	Path cost	Real contigs	Calculated contigs	Correct overlaps	Incorrect overlaps	Accuracy
covid19	GOT	524	0	0	52	0	1.00
	DWVRP	756	0	2	50	0	0.96
lambda_phage	GOT	99	1	5	85	0	0.96
	DWVRP	174	2	9	81	0	0.92
NC_000913_cov5	GOT	282	2	1	45	1	0.98
	DWVRP	518	5	7	40	0	0.96
NC_000913_cov10	GOT	282	1	2	90	0	0.99
	DWVRP	678	4	6	85	1	0.96
NC_000913_cov15	GOT	229	1	1	137	0	1.00
	DWVRP	526	3	10	128	0	0.95

## Discussion

The aim of this study, which was to test the method proposed by Jugas et al. [12] and evaluate its operation in the classical and hybrid (using quantum annealer calculations) computational model, has been achieved. The experiments carried out for both randomly generated and actual DNA data (using actual genomic sequences and reads from the simulator) confirmed the usefulness of the overlaps detection algorithm based on the Pearson correlation coefficient, as well as the proposed method of dividing the path obtained by the travelling salesman algorithm into coherent fragments of DNA sequences (contigs) in the de novo assembly task. The high efficiency of the algorithm was demonstrated for sequences with sufficient coverage and low error content.

The presented algorithm fits in with the approach of searching for the most accurate (error-free) contigs, where we accept obtaining a set of consistent genome fragments instead of one resulting sequence, not necessarily covering the entire genome of interest. By appropriately selecting the threshold of the evaluation function for the algorithm that detects false overlaps, we can obtain high-quality contigs with a low (or even zero) probability of an error. This approach can be used, for example, in the case of assembling genomes of large organisms, where we are primarily interested in coding regions. In addition, the use of alternative forms of DNA sequence representation (other than cumulative phase representation) would be worth considering.

The presented research is preliminary, as this work - as already mentioned—is a proof of concept. It is necessary to conduct experiments for a more significant number of test cases of organisms that differ in terms of belonging to a specific kingdom or phylum and genome size and verify the scalability of the method. In addition, the study would be worth extending to sequences obtained by other sequencers, such as Oxford Nanopore (utilising long-read sequences similar to the tested PacBio). The problem of repeated regions in DNA sequences should also be solved, e.g. by appropriate methods of filtering out erroneous reads (as proposed in [17]).

Several improvements can be made in the implementation of the developed pipeline. It would be worth considering optimising part of the calculations performed on a classic computer, e.g. parallelisation using GPU. In addition, the part responsible for finding the travelling salesman using quantum annealer was based on a ready-made tool dedicated to VRP, not TSP. Willing to apply the discussed method in practice, it would be worth developing a dedicated tool optimised for TSP (as a particular case of VRP).

In order to be able to state whether the computations on quantum annealer—quantum or hybrid—applied to de novo assembly task compete with the classical ones, further research is needed. Since the presented algorithm can by no means be considered a complete assembler, it is difficult to compare the obtained results with the existing tools. In order to be able to actually compare the results obtained with the use of the hybrid approach (QPU + CPU) with classical algorithms, an appropriate method of evaluating the results should be developed, taking into account the specificity of calculations made on the QA. Besides, as it was mentioned in Preliminary assumptions section, a few simplifying assumptions were made in the conducted experiments—the next necessary step in further research is their removal or at least easing of the restrictions. The algorithm would also need to be scaled up in order to be able to analyze genomes larger than viral ones. Only then will it be known whether we should wait for more computing power or whether the current version of quantum annealers can be helpful in practical applications. It should be remembered that quantum computers are still immature in hardware and the number of dedicated algorithms available, be it for quantum annealer or general-purpose quantum computers. In the case of the latter, the gap between theoretical possibilities and hardware development is even more comprehensive. If the computing power would allow the travelling salesman problem to be embedded for thousands of vertices (and not several as today), the possibilities offered by the quantum annealing algorithm could be used directly. For now, however, we are forced to use hybrid methods for problems of the size we have for the de novo assembly issue.

In our opinion, a worth exploring direction is a completely different approach to the de novo assembly problem, i.e. the use of overlap-layout-consensus graphs and an attempt to assemble—by solving the TSP problem-sequences on quantum annealer using the fact that these are sparse, bounded degree graphs. The question posed in this way should be formulated as QUBO. The number of variables necessary for quantum annealer to be considered will be many times lower than for the complete graph.

## Conclusions

The effectiveness of the algorithm proposed by Jugas et al. [12] for artificial and actual DNA sequences was confirmed. The usefulness of quantum annealer for solving the travelling salesman problem was tested for actual sequences (with simulated reads). The algorithm was scaled to the size of viral genomes.

We developed a new algorithm for de-novo assembly that uses both: quantum computer (quantum annealer) and classical electronics computer (CPU). The linear complexity parts of our algorithm are deployed on CPU, the parts with higher complexity on QA. We tested this algorithm on real data, and we used D-Wave VRP implementing hybrid approach.

Our research confirms that computing with the use of quantum annealer can be an alternative to other methods, such as large computing clusters or parallelisation with the use of GPU. This direction is worth exploring by developing dedicated algorithms based on the quantum paradigm.

Our goal was to show a new computational paradigm, using quantum annealing, to solve the practical problem of de novo assembly. Indeed, the results achieved by our algorithm are (in most cases) slightly worse than those achieved by the classical algorithm. However, since these differences are small, it can be assumed after further work on the development of the described method (such as the selection of appropriate processing parameters for QPU strictly for the assembly task, as the current experiments were carried out for the default values used in the D-Wave-VRP tool) and, above all, with the development of QA technology, the chances of these results will be better.

## Data Availability

The code and data used in the presented research are publicly available in the GitHub repository (recent version) and Zenodo (archived version) under the MIT license. The list of NCBI accession numbers of sequences used for this study (dataset B) is available on the GitHub repository.

## Abbreviations

DWVRP: D-Wave-VRP [20] tool
GOT: Google OR Tools
NGS: Next generation sequencing
QA: Quantum annealer
QPU: Quantum processing unit
QUBO: Quadratic unconstrained binary optimization
TSP: Travelling Salesman Problem
VRP: Vehicle Routing Problem

## Footnotes

1 OpenSource, available at GitHub under the Apache License 2.0.
